# AfCHIL, a Type IV Chalcone Isomerase, Enhances the Biosynthesis of Naringenin in Metabolic Engineering

**DOI:** 10.3389/fpls.2022.891066

**Published:** 2022-05-18

**Authors:** Huanhuan Xu, Yanping Lan, Jiayi Xing, Yi Li, Lecheng Liu, Yongqin Wang

**Affiliations:** ^1^Institute of Vegetable Science, Beijing Academy of Agriculture and Forestry Sciences, Beijing, China; ^2^College of Horticulture and Gardening, Yangtze University, Jingzhou, China; ^3^Institute of Forestry and Pomology, Beijing Academy of Agriculture and Forestry Sciences, Beijing, China; ^4^Department of Horticulture, College of Agronomy, Shihezi University, Shihezi, China

**Keywords:** biosynthesis naringenin, chalcone synthase, chalcone isomerase, *Allium fistulosum*, flavonoid

## Abstract

Naringenin is an essential precursor for all flavonoids, and effectively promoting naringenin production is crucial in metabolic engineering. The interaction between plant metabolic enzymes ensures metabolic flux. The effect can effectively improve the natural product synthesis of engineering microbial systems. In this study, chalcone isomerase genes in *Allium fistulosum* have been identified. The expression of AfCHIL is closely related to the accumulation of anthocyanins, and the expression of AfCHIL and AfCHS was highly synchronized. Yeast two-hybrid and firefly luciferase complementation imaging assay further confirmed AfCHIL physically interacted with AfCHS/AfCHI. The bioconversion experiment confirmed that AfCHIL reduced the derailment produced by AfCHS and increased the yield of naringenin. In addition, a system of biosynthesis naringenin involved in AfCHS was constructed, and these results suggested that the potential function between CHS with CHIL advanced naringenin production effectively. In conclusion, this study illustrated the function of AfCHIs in *Allium fistulosum* and provided new insight into improving the synthesis efficiency of naringenin.

## Introduction

**Figure 1:**
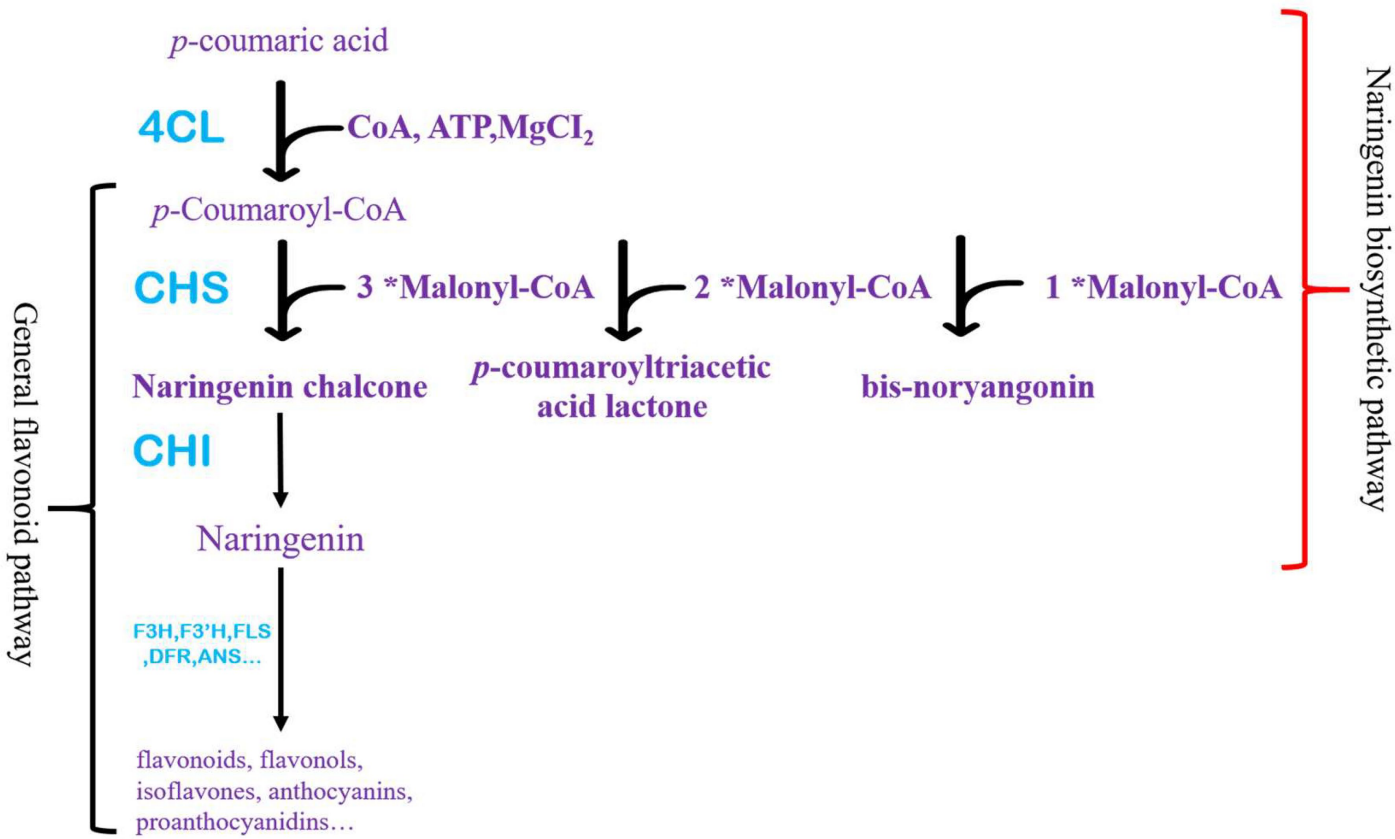
Naringenin and flavonoids pathway in plants. Red brackets represent the naringenin synthesis pathway starting from *p*-coumaric acid, and black brackets generally represent flavonoid synthesis pathways in plants.

Flavonoids have received increased attention across many disciplines in recent years (Nuutila et al., 2003; Agati et al., 2012; Imran et al., 2021). It is the main component of health products, which promotes human health (Barfoot et al., 2021; Ponte et al., 2021). It can effectively prevent cancer, antioxidant, and other physiological effects (Barfoot et al., 2021; Ponte et al., 2021; Sebastian et al., 2021; Parmenter et al., 2021a,b). Flavonoids are widespread in plants and play a significant role (Winkel-Shirley, 2002; Brunetti et al., 2018; Zhao et al., 2019; Ono et al., 2020). They were divided into flavonoids, flavonols, isoflavones, anthocyanins, and proanthocyanidins (Waki et al., 2020). Although each flavonoid is synthesized from different branches of the biosynthetic pathway and regulated separately, naringenin is a precursor of all flavonoids (Ferrer et al., 1999; Ralston et al., 2005). Chalcone synthase (CHS) catalyzes the production of naringenin chalcone, and chalcone isomerase (CHI) ensures the rapid cyclization of naringenin chalcone (Figure 1).

*Allium fistulosum* is a traditional vegetable in Asia and rich in flavonoids. Previous studies have shown that it accumulates massive flavonols (Masuzaki et al., 2006b; Abdelrahman et al., 2019). Different flavonols have been identified, of which quercetin derivatives are the most common (Aoyama and Yamamoto, 2007). *Allium fistulosum* also accumulates anthocyanins, such as anthocyanin 3-glucoside (Abdelrahman et al., 2019). In the report of *A. fistulosum*-*A. cepa* monosomic addition lines, several structural genes involved flavonoid pathway have been identified. CHS-A and CHS-B are located on different chromosomes. Both CHS-A and CHS-B exist in *A. cepa*, but only CHS-B exists in *A. fistulosum* (Masuzaki et al., 2006a,b). Both CHI and CHI-ACAD exist in *A. fistulosum* and *A. cepa.* These EBGs (Early biosynthesis genes) are essential for the efficient synthesis of flavonoids. However, the enzymatic properties of these EBGs in *Allium* have not been reported. The potential relationship between them is even more a mystery.

Naringenin production is mainly through plant extraction, with certain limitations, such as the source of plant materials, the stability of extraction methods, and harmful materials produced (Zang et al., 2019). In addition, the metabolic synthesis of naringenin has been widely studied (Koopman et al., 2012; Liu et al., 2017; Ibdah et al., 2018; Mark et al., 2019; Zhang et al., 2021). 4-coumarin-CoA ligase (4CL), CHS, and CHI are pivotal in synthesizing naringenin. At present, the primary way to improve the efficiency of heterologous synthesis of naringenin is to regulate the expression of critical enzymes, the activity of critical enzymes, and reaction conditions (Ibdah et al., 2018; Gao et al., 2020a,b; Li et al., 2020). Although the by-product produced by CHS (CTAL, *p*-coumarin triacetactone lactone and BYN, bis-noryangonin) was not detected in plants, the by-product produced by CHS derailment had been confirmed (Figure 1). The production of these by-products undoubtedly harms the synthesis of naringenin. Therefore, reducing the by-products is an essential method to synthesize naringenin efficiently (Dastmalchi, 2021).

This study identified the CHIs in *A. fistulosum* and analyzed their enzymatic functions, elucidating the potential functions revealing the molecular mechanism involved in the efficient synthesis of flavonoids. In addition, we established the synthesis system *in vitro* based on the synthesis of naringenin in *A. fistulosum* and optimized the synthesis system of naringenin by type IV CHI in *A. fistulosum*. This study provided an insight into the optimization of metabolic engineering.

## Materials and Methods

### Chemicals

Cyanidin Chloride and Coenzyme A were from Sigma-Aldrich. Naringenin was from Solarbio. Naringenin chalcone, *p*-Coumaryl CoA, and malonyl CoA were from Yuanye Bio-Technology, China. *p*-coumaric acid was obtained from Rhawn, China.

*Allium fistulosum* materials used in this study were grown at the Vegetable Research Farm, Beijing Academy of Agriculture and Forestry Sciences. It is divided into *A. fistulosum* containing anthocyanins (R) and without anthocyanins (W), as shown in Supplementary Figure S1.

### RNA Isolation and Transcriptome Sequencing

*Allium fistulosum* was collected from Beijing, China. Total RNA has been extracted about the method by Yang (Liu et al., 2016). Transcriptome sequencing was performed by Beijing Biomarker Technologies based on the Illumina HiSeq2000 sequence platform. The transcription level detected was estimated by FPKM.

### cDNA Synthesis and RT-PCR

Remove genomic DNA, synthesize cDNA, and Real time-PCR analysis was performed according to the previous protocol (Liu et al., 2014). Each quantitative PCR experiment had three independent biological replicates. The primers used in this study are in Supplementary Table S3.

### Anthocyanin Measurement

The methods for the determination of anthocyanins refer to Sangkyu’s method (Park et al., 2017). An HPLC system (*Agilent 1260 Infinity II*) equipped Poroshell 120 EC-C18 ODS column (2.7 μm 4.6 × 50 mm). Anthocyanins detected with acetonitrile and 0.1% formic acid aqueous solution. Gradient separation of compounds at a flow rate of 300 μl/min: 0–2 min, 2% A; 2–5 min, 2–5% A; 5–10 min, 5% A; 10–15 min, 5–10% A; 15–20 min, 10–20% A; 20–25 min, 20–60% A; 25–30 min, 90–100% A; 30–35 min, 100–20% A; 35–40 min, 2% A. Anthocyanin was detected at 520 nm.

### Genes Isolation and Plasmid Construction

The complete ORF (Open Reading Frame) of *Af4CL*, *AfCHIL*, *AfCHI*, and *AfCHS* was amplified from *A. fistulosum* and cloned into a pMD™18-T vector (TaKaRa) for resequencing. For the expression of recombinant proteins, *AfCHIL*, *AfCHI,* and *AfCHS* were cloned into the pET-15b vector, and then these plasmids transformed into *Escherichia coli* BL21 (DE3). For Y2H assays, *AfCHIL, AfCHS,* and *AfCHI* were cloned into pGADT7 and pGBKT7 vectors, respectively. For firefly luciferase complementation imaging assays, *AfCHIL*, *AfCHI*, and *AfCHS* were cloned into *Pro35S*:*nLUC* and *Pro35S*:*cLUC* vectors.

### Expression and Purification of His_6_-AfCHS, His_6_-AfCHI, and His_6_-AfCHIL

The proteins were expressed according to the method of Rong (Ni et al., 2020). In short, bacterial *fluid* transformed with the corresponding plasmid shook at 37°C, 200 rpm until reaching an OD_600_ of 0.6, then IPTG was added at a final concentration of 0.5 mM. The bacterial culture was cultured for 20 h at 16°C. The protein was purified according to the method provided in Capture™ His-Tagged Purification Kit (TaKaRa). The replacement buffer and storage method are consistent with those described by Zhao et al. (2021).

### Condition of Enzyme Reaction

The purified protein was quantified using a Protein Quantitative Kit (BCA; Trans Gen Biotech, Beijing, China). For the chalcone isomerase assay, the reaction was in a volume of 200 μl, containing 50 mM Tris-HCl (pH 7.5), 2 mM DTT, 10 μg purified protein, and 100 μM naringenin chalcone. The substrate is finally added to the reaction and the reaction incubated for 1 min at room temperature. For chalcone synthase assay, the reaction was in a volume of 250 μl, containing 100 mM HEPES-KOH buffer (pH 7.0), 2 mM DTT, 10 μg purified protein, 200 μM malonyl CoA and *p*-Coumaryl CoA at a series of concentrations (20–200 μM). The reaction was carried out at 35°C for 30 min. For CHS/CHIL complex analysis, the reaction was carried out according to the procedure described by Ban (Ban et al., 2018). After extraction, the product detected at 280 nm. Three biological replicates were analyzed of each enzyme reaction.

### Condition of LC-MS

LC-MS was performed on Waters Acquity UPLC H-Class equipped with Waters SQ Detector 2. The chromatographic separation equipped a C18 column (C18 2.1*50 mm, 1.7 μm). The column temperature was maintained at 40°C. The scan range was operated with the mass *m*/*z* 50–2,000 Da. The elution of the column is consistent with the HPLC system.

### Y2H Assays

The corresponding plasmid was transformed into yeast strain Y187. Positive transformants were selected on SD/-Trp/-Leu medium. To confirm the interaction, the suspension of positive transformants was applied to S.D./- his/-Leu/-Trp medium incubation at 30°C. Photographs were taken after 72 h.

### Firefly Luciferase Complementation Imaging Assay

*Agrobacterium* tumefaciens strain GV3101 containing luciferase structure were mixed and injected into *Nicotiana benthamiana* leaves. The luciferase activity was detected after transfection 48 h. 0.2 mM luciferin treated the transfection leaves and culture in the dark for 15 min.

### Production of Naringenin in *Escherichia coli* Cells

pET-15b-*Af4CL* (negative control), pET15b-*Af4CL*-*AfCHS*-*AfCHI* (CHIL−), and pET15b-*Af4CL*-*AfCHS*-*AfCHI-AfCHIL* (CHIL+) were obtained by enzyme digestion and plasmid recombination recombinant plasmids, respectively (The construction method is shown in Supplementary Figure S4). These plasmids were used to transform *E. coli* strain Rosetta2 (DE3).

The overnight culture was transferred to 20 ml of a liquid *Luria-Bertani* medium and incubated at 37°C until reaching an OD_600_ of 0.6. IPTG was added to the culture to a final concentration of 1 mM, incubated at 16°C for 20 h. The cell suspended in M9 medium for an OD_600_ of 0.5 in a volume of 500 μl contained 2.5 mM *p*-coumaric acid, 5 mM MgCl_2_, 2.5 mM ATP, 200 μM, and 0.6 mM malonyl-CoA. The culture was incubated for 30 h at 26°C. After incubation, the products in the supernatant were extracted with ethyl acetate twice. The residue was dissolved in 100 μl of MeOH for HPLC analyses as described above.

## Results

### Identification CHIL-Fold Protein Family Genes in *Allium fistulosum*

**Figure 2:**
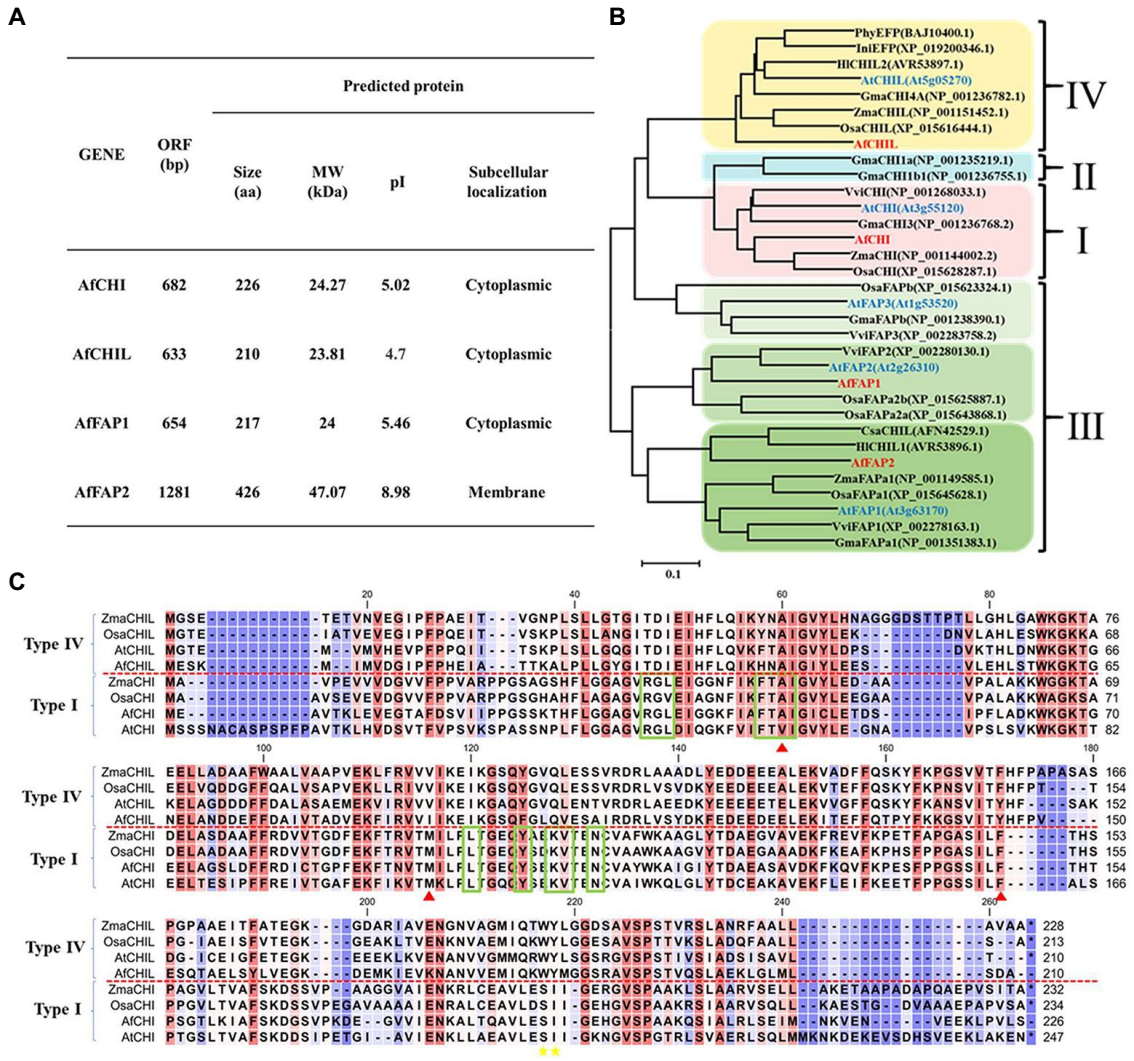
Molecular characteristics of AfCHIs proteins in *Allium fistulosum*. **(A)** Information of chalcone isomerase genes in *A. fistulosum*. **(B)** Phylogenetic analysis of AfCHIs proteins from *A. fistulosum* and other plants. The tree was constructed by MEGA6 based on the multiple sequence alignment conducted by ClustalW and using the Neighbor-joining method. **(C)** Sequence alignment of *A. fistulosum* type I and type IV CHIs with others from *Arabidopsis thaliana*, *Oryza sativa*, and *Zea mays*. The green box represented the key residues of (*2S*)-naringenin binding cleft, respectively. Red triangles indicated the hydrogen bond network in the active site.

To identify CHIL-fold protein family genes in *A. fistulosum*, the full-length transcriptome of *A. fistulosum* was a blast with TBLASTX. AtCHIs, ZmCHIs, and OsCHIs were used as reference sequences. The blast results showed that four CHIL-fold protein genes were in *A. fistulosum*, and the basic features of these genes are described in Figure 2A. Prediction of subcellular localization results suggested that AfCHI and AfCHIL were in cytoplasm. Subcellular localization experiments indicated that AfCHIL was localized to the cytoplasm and nucleus. It was worth noting that AfCHS was the same subcellular localization with AfCHIL (Supplementary Figure S4). Phylogenetic analysis suggested that the CHIL-fold protein genes are divided into three subfamilies. AfCHI belongs to type I CHIs, and AfCHIL was type IV CHIs. AfFAP1 and AfFAP2 are fatty acid-binding proteins and belong to type III CHIs (Figure 2B). CHIs of the same branch shared an overall sequence similarity of 60% (Supplementary Table S1). Furthermore, *AfCHIL* and *AfCHI* were compared by multiple sequence alignment, and the results showed that AfCHI contained the key residues of (*2S*)-naringenin binding cleft, the hydrogen bond network in the active site and the residues of determined substrate preference. However, all the residues lacked in AfCHIL (Figure 2C).

### CHIL-Fold Protein Family Genes Involved in Flavonoids Pathway

**Figure 3:**
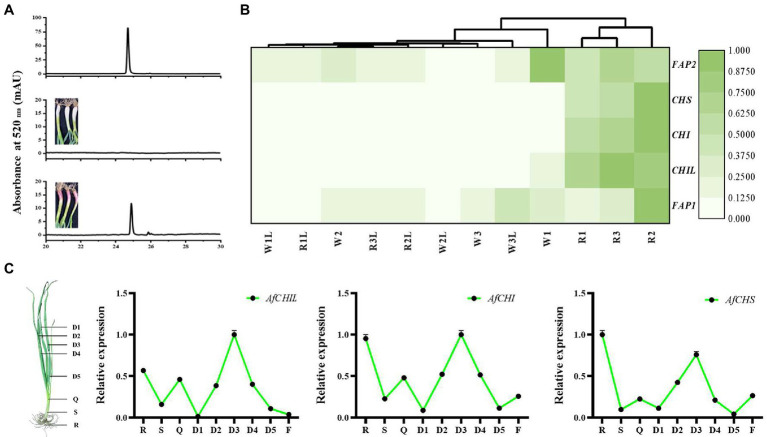
AfCHIs involved in flavonoid pathway of *Allium fistulosum*. **(A)** Analysis of anthocyanin content between W and R. **(B)** Transcriptome analysis of the effect of CHS and CHIs on anthocyanin accumulation. W1, W2, and W3 were stems of W, W1L, W2L, and W3L were leaves of W. R1, R2, and R3 were stems of R. R1L, R2L, and R3L leaves of R. **(C)** Expression of CHS and CHIs in different tissues of *A. fistulosum.* D1(the first leaf), D2(the second leaf), D3(the third leaf), D4(the fourth leaf), D5(the fifth leaf), Q(sheath), S(stem), R(root), and F(flower) were used for expression analysis.

To confirm AfCHIs genes involved in flavonoid biosynthesis, the stems and leaves of two materials of *A. fistulosum* were analyzed (Supplementary Figure S1). HPLC analysis results showed that anthocyanins accumulated in the stems of R (Figure 3A). RNA-Seq analysis suggested that the expression of EBGs in R was much higher than W (Figure 3B). It is worth noting that the expression of AfCHIL is much higher than AfCHI (Supplementary Table S2). These results suggested that the expression of those EBGs was closely related to the accumulation of anthocyanins and flavonoids. Further qRT-PCR suggested that CHS and CHIs in *A. fistulosum* had the nearly same expression pattern (Figure 3C). In summary, these results indicated that these EBGs in *A. fistulosum* genes have the same biosynthetic process.

### Enzyme Activity Assays of AfCHIs Recombinant Protein

**Figure 4:**
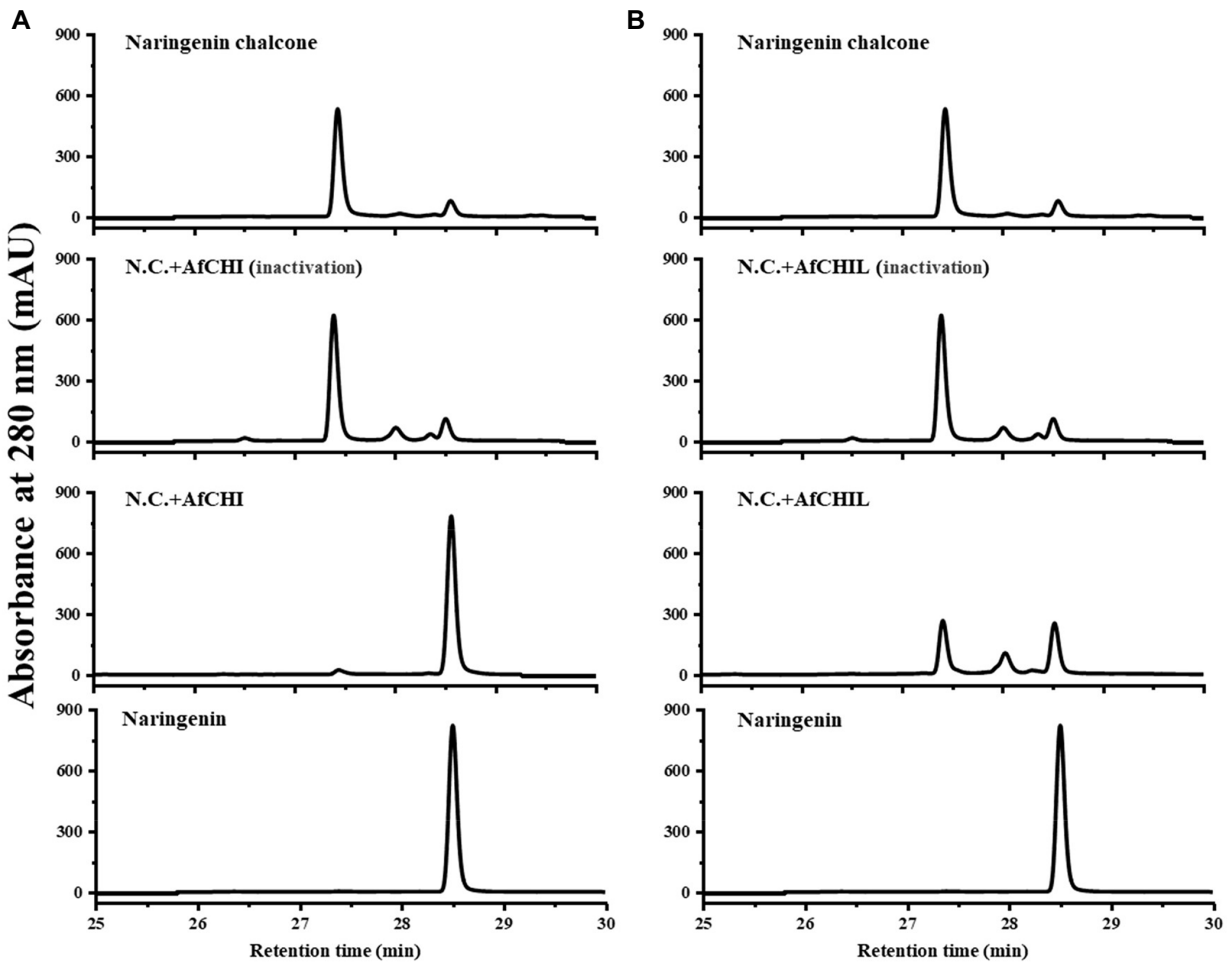
*In vitro* enzymatic assays of the recombinant AfCHI and AfCHIL. **(A)** HPLC analyses of AfCHI reaction products. **(B)** HPLC analyses of AfCHIL reaction products. The assays were conducted with naringenin chalcone as substrate, and 10 μg protein was added in each reaction. N.C., naringenin chalcone; N.A., naringenin; AfCHI/CHIL (95°C), AfCHI/CHIL treatment at 95°C for 15 min.

Two CHIs genes in *A. fistulosum* were isolated and expressed in *E. coli* using pET-15b, and the recombinant proteins were purified. SDS-PAGE analysis showed that the actual molecular weight of the purified recombinant protein was consistent with the predicted value (Supplementary Figure S2). The catalytic activity of the recombinant enzyme was analyzed with naringenin chalcone as a substrate. The catalytic products were analyzed by HPLC with naringenin as standards. The results showed that AfCHI had the catalytic activity on naringenin chalcone (Figure 4A). Compared with the control, AfCHIL can catalyze the formation of naringenin slowly, although this efficiency is much slower than the spontaneous cyclization of naringenin (Figure 4B). These results suggested that CHI had the unique catalytic ability of CHI type I and was the real CHI in *A. fistulosum*.

### AfCHIL Physically Interact With AfCHI and AfCHS

**Figure 5:**
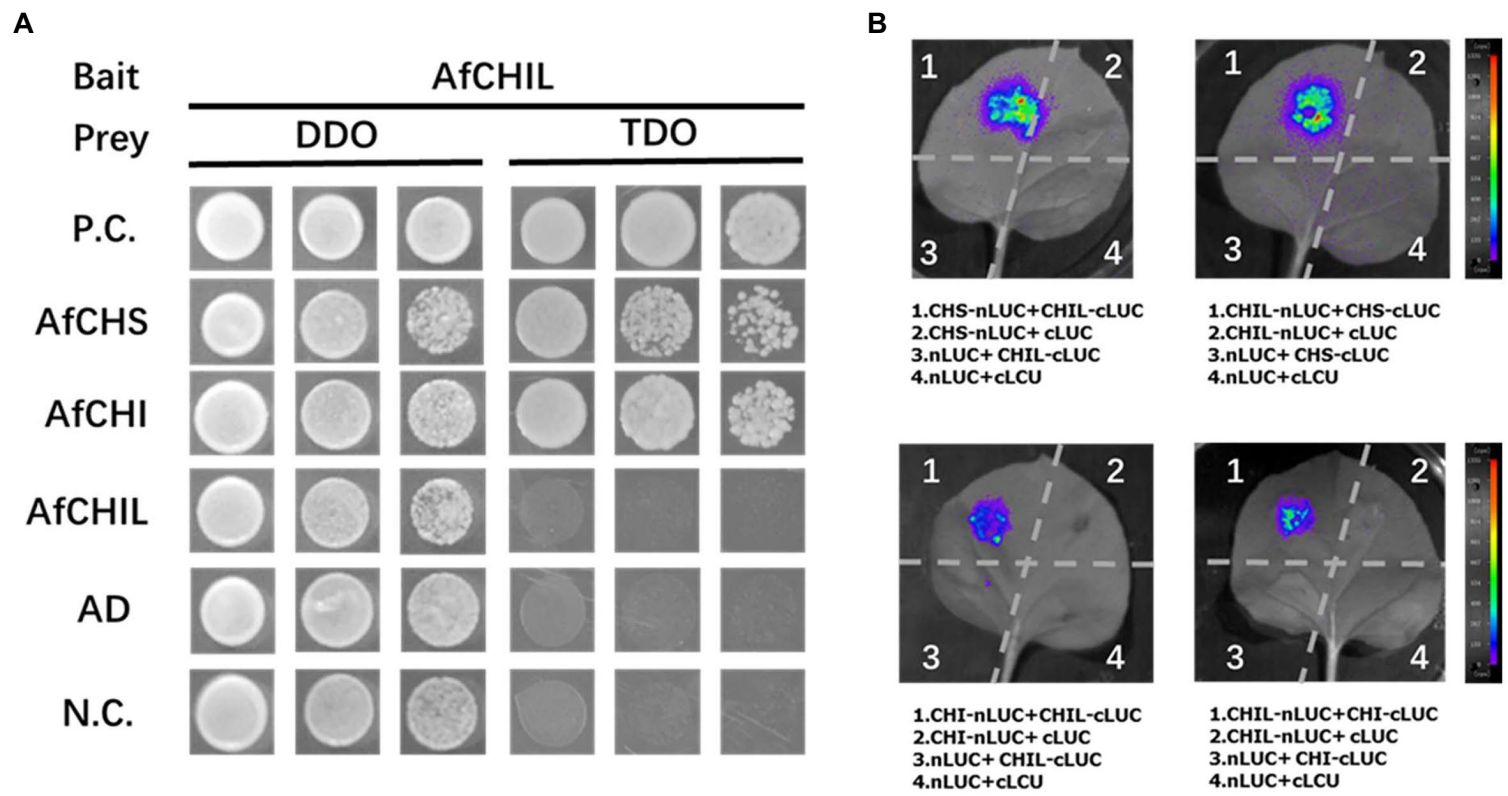
Interaction between AfCHIL, AfCHS, and AfCHI. **(A)** Yeast two-hybrid experiment demonstrating the protein-protein interaction between AfCHIL, AfCHS, and AfCHI. The TDO medium (SD-Trp-Leu medium) and TDO (SD-Trp-Leu-His medium) medium were used as selective media. P.C. refers to the positive control; N.C. refers to the negative control. **(B)** Luciferase complementation imaging assay was used for analyzing the protein–protein interaction of AfCHIL, AfCHS, and AfCHI. Agrobacterium tumefaciens carrying different plasmids were infiltrated into *Nicotiana benthamiana* leaves. Images were recorded at 72 h after infiltration.

The interaction between AfCHIL and AfCHS or AfCHI was analyzed by the Y2H system. The results showed that AfCHIL and AfCHS co-transformed yeast could grow on the medium lacking-Trp, -Leu, and -His. In addition, yeast growth was also detected when AfCHIL was co-transformed with AfCHI. However, no significant yeast growth was detected in the negative control (Figure 5A). These results suggest that there is a protein–protein interaction between AfCHIL and AfCHS or AfCHI. LCI analysis was used to further examine the binary protein–protein interaction of the above combination in plants. When it was used for AfCHIL/AfCHS, this method could obtain a strong fluorescence signal. In AfCHIL/AfCHI, relatively weak fluorescence signals can also be detected (Figure 5B). However, AfCHI/AfCHS is co-expressed in tobacco, and no fluorescence signal can be detected regardless of the position of the protein at the N-terminal or C-terminal in Supplementary Figure S3. LCI and the results of the Y2H system confirmed that AfCHIL could participate in the biosynthesis of flavonoids in *A. fistulosum* through the interaction with AfCHIL/AfCHS.

### AfCHIL, a Type IV CHI-Fold Protein Enhance the Activity of CHS

**Figure 6:**
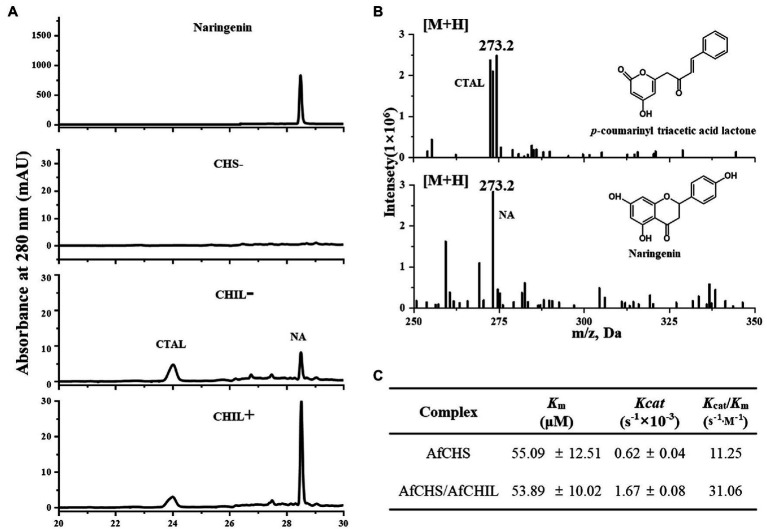
Effect of AfCHIL on enzymatic properties of AfCHS. **(A)** HPLC chromatograms showing the effect of AfCHIL on the enzymatic activity of AfCHS *in vitro*: N.A., naringenin standard; empty control, pET15b empty (CHS−); AfCHS (CHIL−), and AfCHS/AfCHIL(CHIL+). **(B)** LC-MS analysis of CTAL and naringenin. **(C)** Kinetic parameters of AfCHS or AfCHIL/AfCHS1 complexes with *p*-coumaroyl-CoA and malonyl-CoA as substrates. For kinetic determination, malonyl-CoA at a fixed concentration of 200 μM and *p*-coumaroyl-CoA at a series of concentrations (20–200 μM), The *K*_m_ and *K*_cat_ values were calculated by nonlinear regression curves fitted to the Michaelis–Menten equation using GraphPad Prism 8.0.

To confirm the regulatory effect of AfCHIL on AfCHS by analyzing the activity changes of AfCHS on *p*-coumaroyl CoA and malonyl CoA in the presence or absence of CHIL. We expressed the isolated AfCHIL and AfCHS in *E. coli* (Supplementary Figure S2). The product was analyzed by HPLC. Here, two products are generated based on the reaction of AfCHS and AfCHS/AfCHIL. The HPLC retention time of the latter was the same as naringenin standard (Figure 6A). When the reaction only exists AfCHIL, the reaction failed to produce naringin (Supplementary Figure S5). The reactants were further analyzed by LC–MS. The results showed that the former was CTAL (Figure 6B). Regardless of the concentration of *p*-coumaroyl CoA, the reaction using enzyme combination AfCHS/AfCHIL was higher than naringenin using AfCHS combination only. When AfCHIL was present in the reaction, naringin increased by 2.2 times and CTAL decreased by 30% (Supplementary Figure S6).

Further examining the differences in catalyst efficiency for comparison (Supplementary Figure S7), we found that AfCHIL did not affect the *K*_m_ value of AfCHS affinity. It is worth noting that *K*_cat_ is increased by about 2.7 times in the presence of AfCHIL (Figure 6C). These results show that AfCHIL can efficiently produce naringenin through direct interaction with AfCHS to reduce the CTAL produced by AfCHS derailment activity, to make the substrate flow into the flavone pathway better.

### AfCHIL in Biosynthesis of Naringenin From *p*-Coumarin Acid

**Figure 7:**
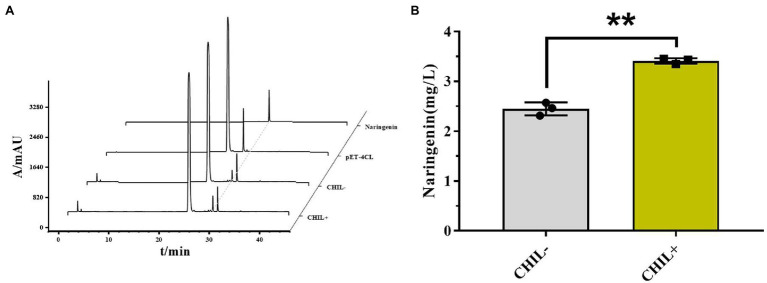
AfCHIL in the biosynthesis of naringenin from *p*-coumarin acid. **(A)** HPLC of catalyzed reactions during the production of naringenin from *p*-coumaric acid in *Escherichia coli* carrying pET15b-4CL (negative control), pET15b-4CL-CHS-CHI (CHIL−) and pET15b-4CL-CHS-CHI-CHIL (CHIL+) are shown. Naringenin, naringenin standard. **(B)** Effect of the presence or absence of CHIL on naringenin synthesis. Data are presented as the average of three independent determinations of three biological replicates (±standard error). ^**^p < 0.01.

To confirm the effects of AfCHIL on producing naringenin in metabolic engineering, the *E. coli* cells were genetically engineered (Supplementary Figure S8). In short, a series of enzymes including Af4CL, AfCHS, and AfCHI involved in naringenin synthesis were expressed in *E. coli*. Western blot was used to analyze the expression of these proteins, the results showed that the protein expression in CHIL− was higher than that in CHIL+ (Supplementary Figure S9). Compared with pET-4CL control, the peak with the same retention time as the naringenin standard can be obtained whether exist CHIL or not (Figure 7A). The results showed that we successfully used *E. coli* to produce naringenin. Although more proteins can be expressed in CHIL−, the production of naringenin increased by 39.24% in the presence of AfCHIL (Figure 7B). In summary, these results showed that AfCHIL could significantly promote the synthesis of naringenin *in vitro*.

## Discussion

The CHI-fold protein family in *A. fistulosum* has been identified in this study. In previous studies, CHI-fold family protein was encoded by a small gene family, which varies from species to species (Ngaki et al., 2012; Przysiecka et al., 2015; Zhu et al., 2021). Four CHI-fold family protein genes have been isolated from *A. fistulosum*. Evolutionary analysis and multiple sequence alignment showed that only type I, type IV, and type III exist in *A. fistulosum*. Type III CHI is fatty acid binding protein devoid of catalytic activities, which is the prototype of other CHI-fold protein. Type II CHI is the transition from type IV to type I, which exists only in ancient plants, liverworts, and spike moss (Ni et al., 2020). In citrus, the expression patterns of CitCHIs and CitCHSs were highly synchronized and the same subcellular localized. In addition, the expression of type IV CHI was higher than that of other CHIs (Zhao et al., 2021). In this study, AfCHIL and AfCHI have the same expression pattern, and the expression of AfCHIL was higher than AfCHI. These results suggest that AfCHIL is a core component to ensure the efficient synthesis of flavonoids in *A. fistulosum*.

*In vitro* experiments also confirmed that Type I CHI can convert naringenin chalcone into naringenin. Our results showed that AfCHI was a real CHI with typical cyclization activity. Type IV CHI is the ancestor of type II CHI protein. It is noncatalytic in many species, including soybean (Dastmalchi and Dhaubhadel, 2015) and *Arabidopsis* (Ngaki et al., 2012). Recent studies on type IV CHI protein show that type IV CHI protein can advance the cyclization of chiral (*S*)-flavanones only in a high concentration of protein (Zhao et al., 2021). In our study, we confirmed that type IV CHI could also bind to chalcone and convert into *s*-isomer. This efficiency is much lower than the self-cyclization rate of chalcone naringenin. In our system, the generated *s*-naringenin can be detected without reaching the concentration mentioned in Zhao’s study (Zhao et al., 2021). Considering the genetic complementarity of the *Arabidopsis chil* mutant, heterologous CHILs cannot complement. The different activities in CHILs may also be one of the factors. We speculate that the binding ability of IV CHIs in different species to naringenin chalcone is different, although this slow activity is difficult to detect.

Previous studies have shown that metabolic enzymes interact to form a structure–function complex to participate in metabolism and ensure effective control of metabolic flow (Jiang et al., 2015; Ban et al., 2018; Ni et al., 2020; Dastmalchi et al., 2021). There is great differences in the metabolic complexes of flavonoids among species. For example, in *Arabidopsis* (Jiang et al., 2015), CHIL and CHI can directly interact with CHS, and there are also interactions between CHS and CHIL in rice (Waki et al., 2020). However, CHI cannot interact with CHIL and CHS to form a complex. In addition, this interaction forms a complex and binds with proteins anchored to the endoplasmic reticulum, such as F3’H and FNS, ensuring the efficient metabolic flux of flavonoids. In this study, the interaction between CHIL-CHS and CHS-CHI was confirmed in *A. fistulosum*. The expression of CHS in accumulating anthocyanins was much higher than that of CHI and CHIL. These results suggest that CHIL is the core component of EBGs in *A. fistulosum*. CHIL forms a complex through the combination of CHS/CHI, and this complex needs to interact with membrane proteins anchored to endoplasmic reticulum, such as F3’H, to ensure the efficient metabolic flux of flavonoid production.

Four types of CHI have been reported in woody plants, herbs, bryophytes, and ferns, indicating that this mechanism is a strategy of plant evolution (Waki et al., 2020). However, the mechanism by which CHIL regulates catalytic efficiency is unknown. CHS has been shown to interact with CHIL and other enzymes in the biosynthesis of flavonoids and isoflavones to form metabolic multienzyme complexes. Previous studies have shown that the combined output of polyketones increased only slightly, indicating that the activity was corrected rather than enhanced. In our study, when we use *p*-coumaroyl CoA and malonyl CoA as substrates, CHS and CHS/CHIL have the catalytic activity of CTAL. Although there is no significant difference in the catalysis of these two substrates, in the presence of CHIL, the synthetic flux of naringenin increases significantly, while CTAL decreases significantly. These results show that CHIL can change the production of CHS derailment products by binding with CHS, reducing the efficiency of flavonoid biosynthesis, ensuring the downstream flavonoid flux, and participating in the flavonoid biosynthesis.

Flavonoid biosynthesis has been clarified in recent years, but the current discoveries show that the auxiliary components play an important role in flavonoid biosynthesis. These elements can guide the metabolic flow and improve yield. Using these neglected components in engineering microbial systems to synthesize natural products is very important (Waki et al., 2020). In previous studies, CHS used for naringenin biosynthesis mostly comes from plants. Some studies have shown that the interaction between CHS/CHIL is a conservative strategy in the process of plant adaptive evolution (Waki et al., 2020). In this study, the enzymatic properties of AfCHIL were described. We produced naringenin in *E. coli* by using related enzymes in *A. fistulosum*. In the presence of CHIL, the production of naringenin was significantly higher than that of CHIL−. Our studies show that CHIL, an auxiliary component of flavonoids synthesis, has a strong ability to improve the yield in the synthesis of naringenin. To confirm the promoting effect of these auxiliary components in biosynthesis, we constructed a naringenin synthesis system designed for Welsh Onion CHS. The results showed that AfCHIL significantly enhanced the production of naringenin. In the presence of AfCHIL, the yield of naringenin increased by 39.24%. These results show that these auxiliary components are an important optimization part of the metabolic synthesis system.

## Conclusion

In the present study, 4 CHI-fold protein genes were identified from *A. fistulosum*. Transcriptome analysis revealed that AfCHI and AfCHIL are involved in flavonoid and anthocyanin production. Enzyme activity assay indicated that AfCHI is the real chalcone isomerase in *A. fistulosum*, although AfCHIL has low activity on naringin chalcone. Y2H assay and LCI confirmed the interaction between AfCHIL and AfCHI/AfCHS. The production of by-product reduced and the synthesis efficiency of flavonoid improved by AfCHS interacted with AfCHIL. In addition, AfCHIL can effectively improve the synthesis efficiency in the enzyme system for the synthesis of naringin involved in AfCHS. The findings will lay the foundation for the molecular mechanism of efficient synthesis of flavonoids in *A. fistulosum* and provided new insights for improving the efficiency of flavonoids synthesis *in vitro*.

## Data Availability Statement

The data presented in the study are deposited in the NCBI repository, accession number PRJNA827977. The original contributions and material presented are included in the article, further inquiries can be directed to author.

## Author Contributions

HX and YW contributed to the conception of the study. HX and JX performed experiments. HX and YLi contributed significantly to analysis and manuscript preparation. LLiu, YLa, and YW performed the data analyses. HX and YW wrote the manuscript. All authors contributed to the article and approved the submitted version.

## Funding

This work was supported by the National Natural Science Foundation of China (31972409), Reform and Development Project of Vegetable Research Center of Beijing Academy of Agricultural and Forestry Sciences (KYCX10), and Scientific and Technological Innovation Capability Project of Beijing Academy of Agricultural and Forestry Sciences (KJCX20200113).

## Conflict of Interest

The authors declare that the research was conducted in the absence of any commercial or financial relationships that could be construed as a potential conflict of interest.

## Publisher’s Note

All claims expressed in this article are solely those of the authors and do not necessarily represent those of their affiliated organizations, or those of the publisher, the editors and the reviewers. Any product that may be evaluated in this article, or claim that may be made by its manufacturer, is not guaranteed or endorsed by the publisher.
