# Supplementary material for: AfCHIL, a Type IV Chalcone Isomerase, Enhances the Biosynthesis of Naringenin in Metabolic Engineering
Source: Front Plant Sci. 2022 May 18;13:891066. doi: 10.3389/fpls.2022.891066 (PMC9158529; doi:10.3389/fpls.2022.891066)
Supplement: Supplementary file 1 [file Data_Sheet_1.docx]

Supplementary Material

**Figure S1. The plant material of *Allium fistulosum* for transcriptome sequencing analysis.** *Allium fistulosum* on the left called W and *Allium fistulosum* on the right called R.

W is no accumulation of anthocyanins, and the stem is white. R is accumulation of anthocyanins, and the stem is red.


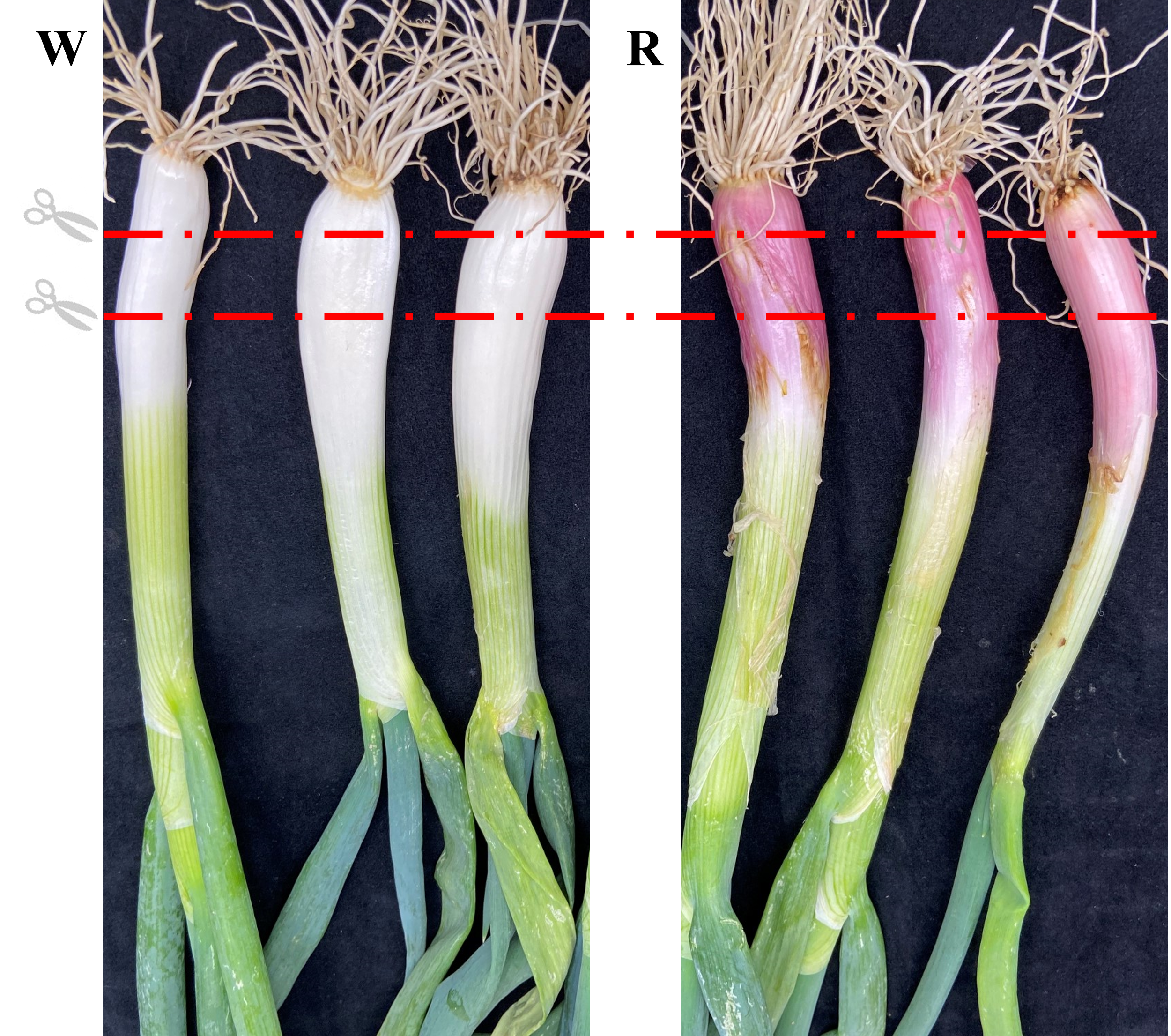


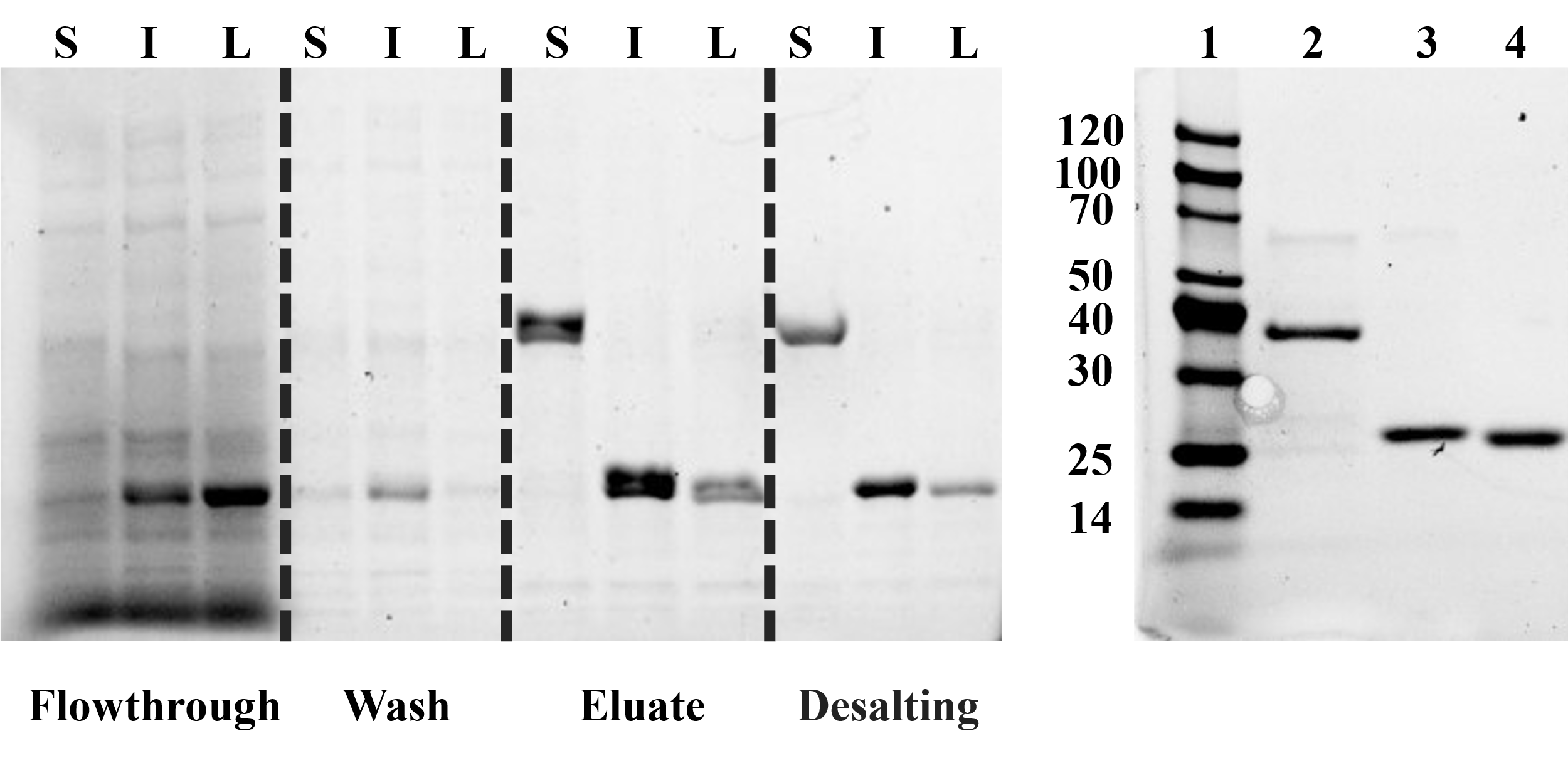


**Figure S2: SDS-PAGE of purified His_6_-AfCHS, His_6_-AfCHI, His_6_-AfCHIL.**

S, His6-AfCHS; I, His6-AfCHI; L, His6-AfCHIL. 1, protein marker; 2, purified His6-AfCHS; 3, purified His6-AfCHI; 4, purified His6-AfCHIL


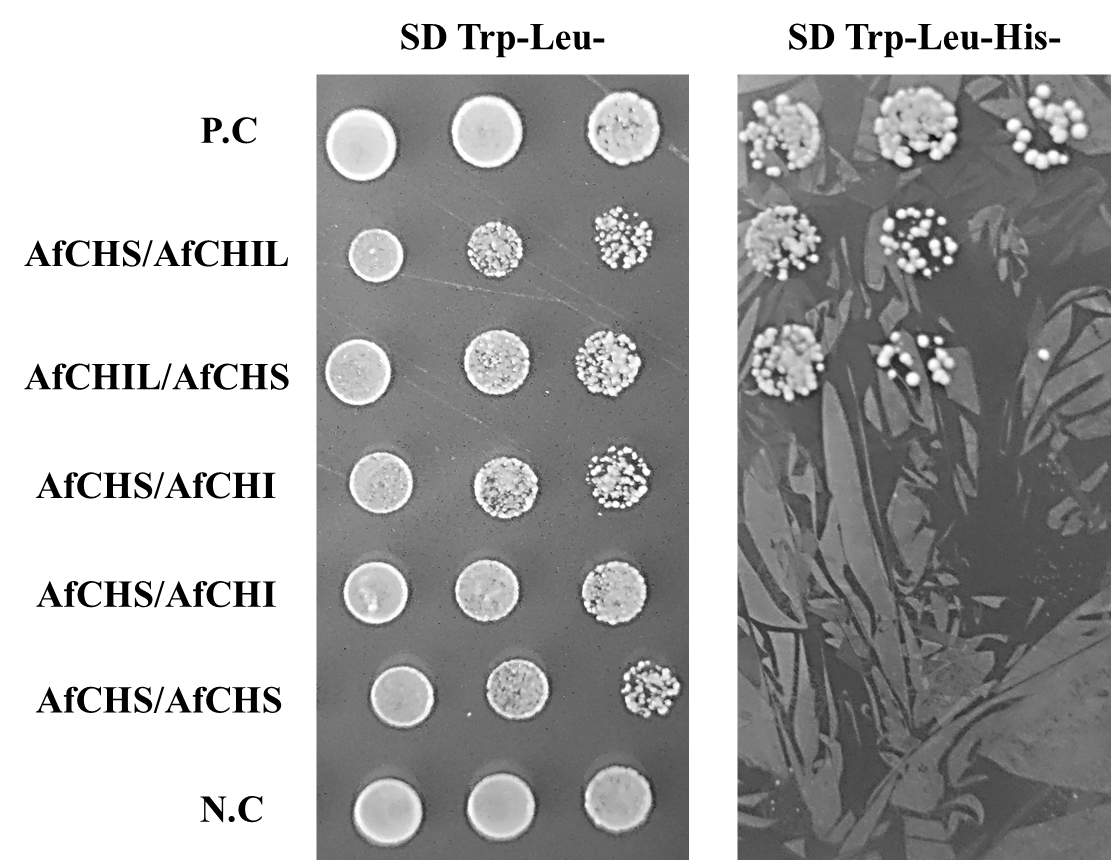

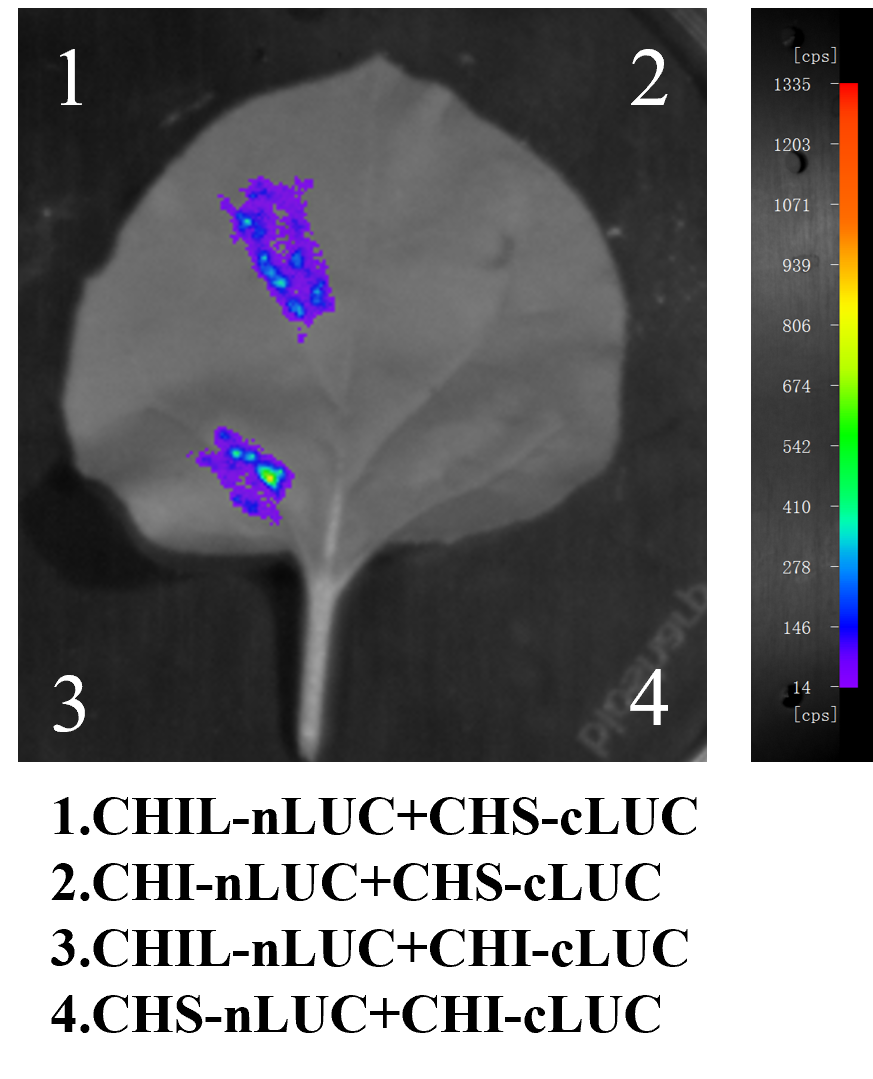


Figure S3. Interaction between AfCHS and AfCHI. (A) Yeast two-hybrid experiment demonstrating the protein-protein interaction between AfCHS and AfCHI. (B) Luciferase complementation imaging assay was used for analyzing the protein-protein interaction of AfCHS and AfCHI.


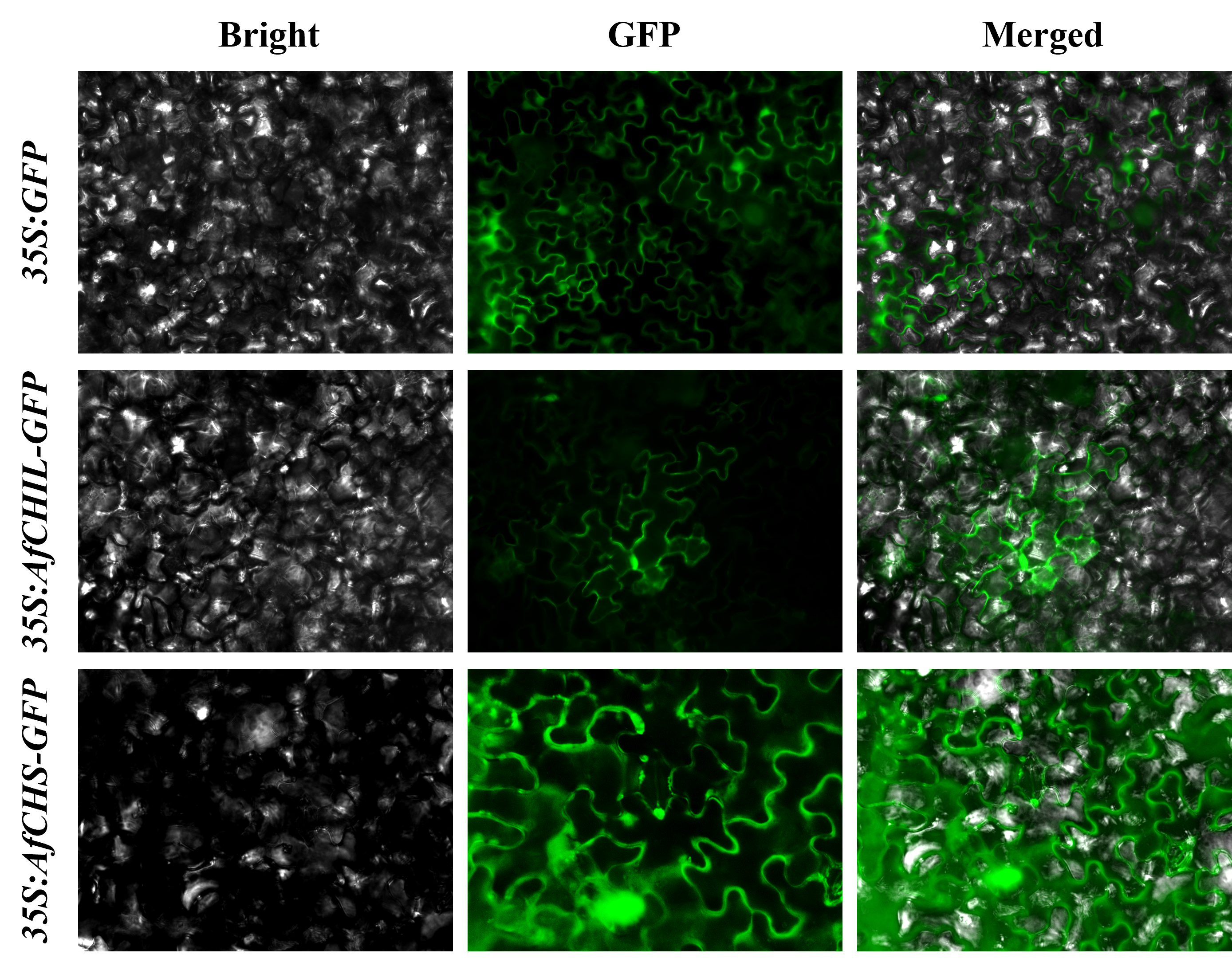


Figure S4 Subcellular localization of AfCHS and AfCHIL. We used Pro35S:GFP empty vector as a control, and fused AfCHS and AfCHIL proteins with GFP to observe their subcellular localization.


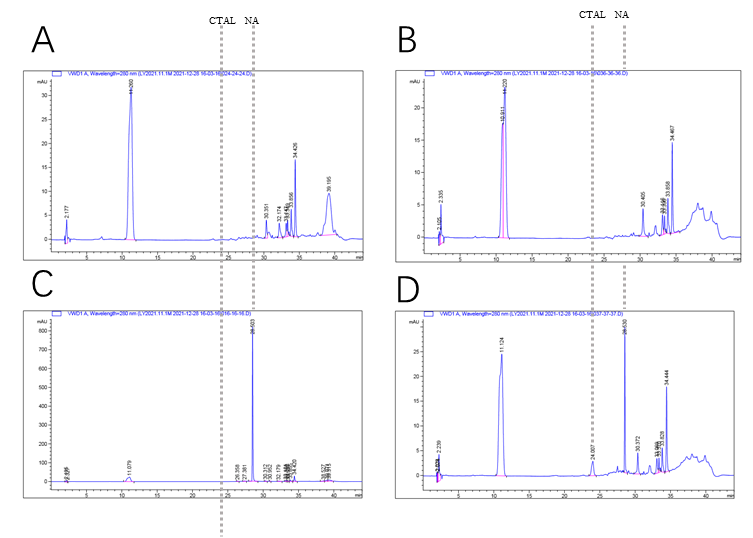


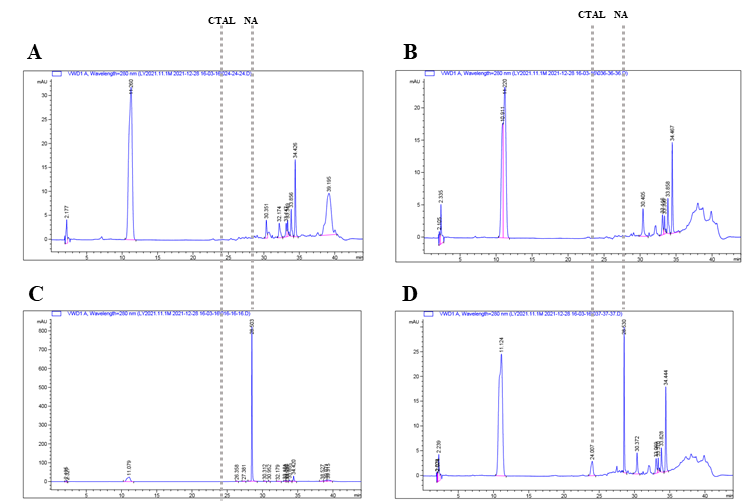


Figure S5 HPLC chromatograms showing the effect of AfCHIL on the enzymatic activity of AfCHS in vitro. (A) empty control, pET15b empty control. (B) only AfCHIL. (C) naringenin standard. (D) AfCHS and AfCHIL


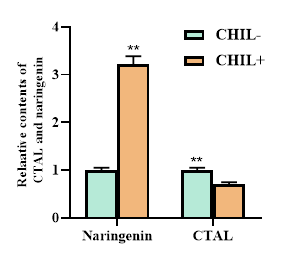


**Figure S6.** Relative contents of CTAL and naringenin produced by AfCHS or AfCHS/AfCHIL. Data are presented in the form mean ±SD (n=3); ***P*<0.01.


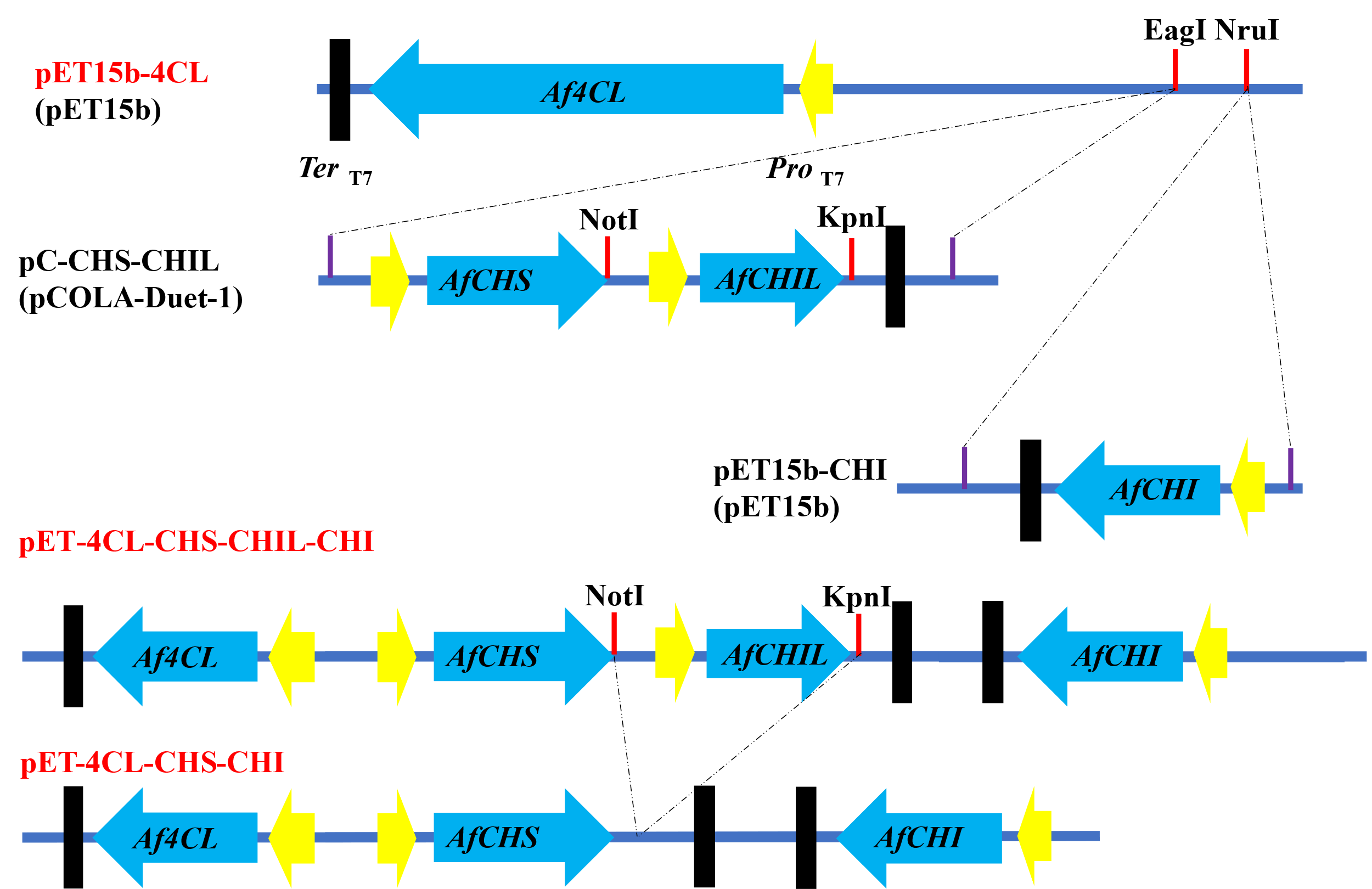


**Figure S8. The constructed Genetically engineered *E.* coli to produce naringenin.** pET15b-4CL，pET15b-CHI, and pC-CHS-CHIL composed pET-4CL-CHS-CHIL-CHI. Removal of AfCHIL using enzyme digestion of NotⅠ and KpnⅠ to generate pET-4CL-CHS- CHI.


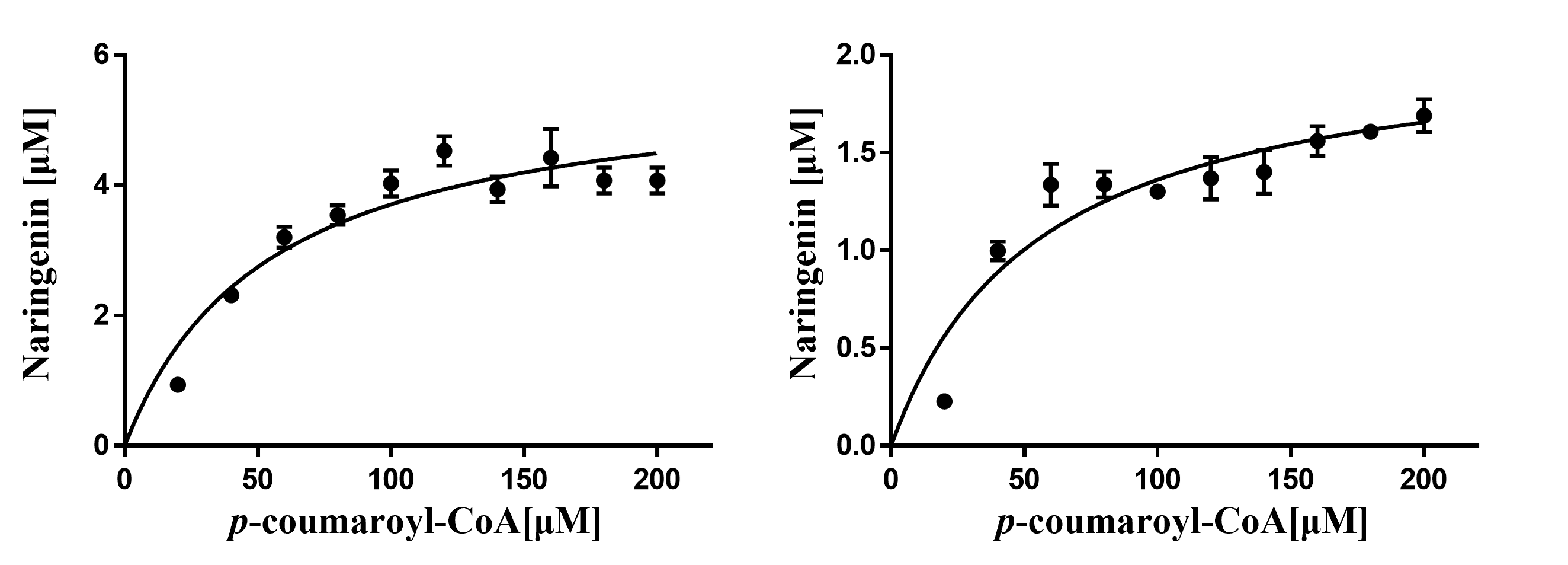


**Figure S7. Michaelis-Menten plots of AfCHS and AfCHS/AfCHIL complex**.

The kinetic parameters Km and Vmax were calculated by nonlinear regression analysis using GraphPad Prism 8 software. These results were averages of three experiments. Error bars denote SE of the means.


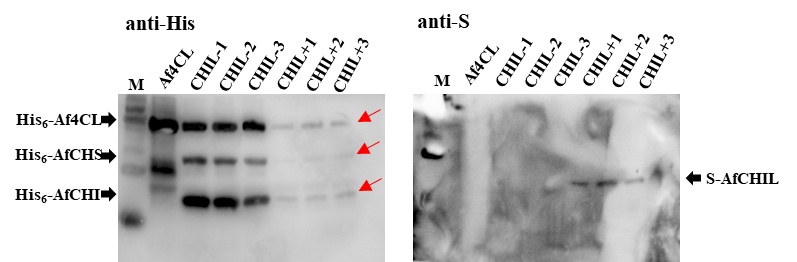


**Figure S9.** Immunological quantification of pET15b-4CL(Af4CL), pET-4CL-CHS-CHI (CHIL–) and pET-4CL-CHS-CHIL-CHI (CHIL+) were produced in the engineered *E. coli* cells. To quantify the expressed Af4CL, AfCHS, AfCHI and AfCHIL proteins, in which Af4CL, AfCHS and AfCHI was His_6_-tagged while AfCHIL was S-tagged, the 30 μL crude extracts of the engineered *E. coli* cells were subjected to SDS-PAGE followed by western blotting analyses using anti-His6 IgG (to determine Af4CL, AfCHS and AfCHI) and anti-S IgG (to determine AfCHIL).

| **Table S1. Identity analysis of CHIs genes from different species.** | | | | | | | |  |
| --- | --- | --- | --- | --- | --- | --- | --- | --- |
|  | ZmaCHIL | OsaCHIL | AtCHIL | AfCHIL | ZmaCHI | OsaCHI | AfCHI | AtCHI |
| ZmaCHIL | - | 69.74 | 55.26 | 52.63 | 28.51 | 28.8 | 23.98 | 23.86 |
| OsaCHIL | 69.74 | - | 62.04 | 58.99 | 28.22 | 27.16 | 25.21 | 23.35 |
| AtCHIL | 55.26 | 62.04 | - | 55.81 | 25.52 | 24.58 | 25.96 | 22.83 |
| AfCHIL | 52.63 | 58.99 | 55.81 | - | 26.25 | 24.9 | 25.53 | 22.35 |
| ZmaCHI | 28.51 | 28.22 | 25.52 | 26.25 | - | 77.12 | 61.44 | 54.4 |
| OsaCHI | 28.8 | 27.16 | 24.58 | 24.9 | 77.12 | - | 61.11 | 52 |
| AfCHI | 23.98 | 25.21 | 25.96 | 25.53 | 61.44 | 61.11 | - | 55.82 |
| AtCHI | 23.86 | 23.35 | 22.83 | 22.35 | 54.4 | 52 | 55.82 | - |

| **Table S2. Transcriptome analysis of EBGs genes in *Allium fistulosum.*** | | | | | | | | |
| --- | --- | --- | --- | --- | --- | --- | --- | --- |
| Gene name | Pfam annotation | W FPKM | WL FPKM | R FPKM | RL FPKM | W: R | W: W.L. | R: RL |
| g536041 | Chalcone-flavanone isomerase | 14.93 | 9.00 | 115.56 | 18.47 | UP | normal | normal |
| g97733 | Chalcone-flavanone isomerase | 4.95 | 10.70 | 211.16 | 13.03 | UP | normal | normal |
| g228276 | Chalcone synthases | 1.54 | 0.50 | 410.12 | 0.71 | UP | normal | normal |

| **Table S3. Primers were used in the present study.** | |
| --- | --- |
| **Primer Name** | **Sequence (5' to 3')** |
| **For gene cloning** | |
| CHS-F | ATGTCAAAGATTGAGGAGAT |
| CHS-R | TCAACCATCAATGGCCACACTCC |
| CHIL-F | ATGGAGTCGAAGATGATCAT |
| CHIL-R | TTAAGCGTCCGATAACATTA |
| CHI-F | ATGGAAGCAGTGACAAAGTT |
| CHI-R | TTATGAAAGCACCGGTAACT |
| 4CL-F | ATGGGTTCAATATCAATGGATC |
| 4CL-R | TCATGGTTGCTGAACATTAGG |
| **For quantitative RT-PCR** | |
| afqchs-f | GTTGTCGAAATCCCAAAGCTC |
| afqchs-r | AAGATGAGGTGGGTTATGTGG |
| afqchil-f | AACATCTGAGCACCTGGAAG |
| afqchil-r | GCAGTCACAATGGCATCAAAG |
| afqchi-f | AGAATAAGGCGCTGACACAG |
| afqchi-r | CCGACAATCTCAATGCTATGC |
| actin-f | ACACGGCCTGGATAGCAACAT |
| actin-r | AGAGCAGTATTCCCAAGCATT |
| **For Y2H assays (into pGADT7 or pGBKT7 vector)** | |
| AD-CHS-F | GGCCATGGAGGCCAGTGAATTCATGTCAAAGATTGAGGA |
| AD-CHS-R | CAGCTCGAGCTCGATGGATCCCCTAACCATCAATGGCCA |
| AD-CHIL-F | GGCCATGGAGGCCAGTGAATTCATGGAGTCGAAGATGAT |
| AD-CHIL-R | CAGCTCGAGCTCGATGGATCCCTTAAGCGTCCGATAACA |
| AD-CHI-F | GGCCATGGAGGCCAGTGAATTCATGGAAGCAGTGACAAAGTT |
| AD-CHI-R | TGCAGCTCGAGCTCGATGGATCCCTCATGAAAGCACCGGTAACT |
| BD-CHIL-F | TATGGCCATGGAGGCCGAATTCATGGAGTCGAAGATGAT |
| BD-CHIL-R | CCGCTGCAGGTCGACGGATCCCTTAAGCGTCCGATAACA |
| BD-CHI-F | TATGGCCATGGAGGCCGAATTCATGGAAGCAGTGACAAA |
| BD-CHI-R | CCGCTGCAGGTCGACGGATCCCTCATGAAAGCACCGGTA |
| **For LCI assays (into nLUC and cLUC vectors)** | |
| CHIL-nLCU-f | GACGAGCTCGGTACCATGGAGTCGAAGATGATCAT |
| CHIL-nLCU-r | ACGAGATCTGGTCGACAGCGTCCGATAACATTA |
| CHIL-cLUC-f | GTCCCGGGGCGGTACCATGGAGTCGAAGATGATCAT |
| CHIL-cLUC-r | AGCTCTGCAGGTCGACTTAAGCGTCCGATAACATTA |
| CHS-nLCU-f | GACGAGCTCGGTACCATGTCAAAGATTGAGGAGAT |
| CHS-nLCU-r | ACGAGATCTGGTCGACACCATCAATGGCCACAC |
| CHS-cLCU-f | GTCCCGGGGCGGTACCATGTCAAAGATTGAGGAGAT |
| CHS-cLCU-r | AGCTCTGCAGGTCGACTTAACCATCAATGGCCACAC |
| CHI-nLCU-f | GACGAGCTCGGTACCATGGAGTCGAAGATGATCAT |
| CHI-nLCU-r | ACGAGATCTGGTCGACTGAAAGCACCGGTAACT |
| CHI-cLUC-f | GTCCCGGGGCGGTACCATGGAAGCAGTGACAAAGTT |
| CHI-cLUC-r | AGCTCTGCAGGTCGACTCATGAAAGCACCGGTAACT |
| **For recombinant protein constructs (into pET15b vector)** | |
| pET-CHS-F | TGCCGCGCGGCAGCCATATGATGTCAAAGATTGAGGAGAT |
| pET-CHIL-F | TGCCGCGCGGCAGCCATATGATGGAGTCGAAGATGATCA |
| pET-CHI-F | TGCCGCGCGGCAGCCATATGATGGAAGCAGTGACAAAGTT |
| pET-CHS-R | TTAGCAGCCGGATCCTCGAGACCATCAATGGCCACACTCC |
| pET-CHIL-R | TTAGCAGCCGGATCCTCGAGAGCGTCCGATAACATTAATC |
| pET-CHI-R | TTAGCAGCCGGATCCTCGAGTGAAAGCACCGGTAACTTTTC |
| pET-4CL-F | TGCCGCGCGGCAGCCATATGATGGGTTCAATATCAATGGA |
| pET-4CL-R | GGGCTTTGTTAGCAGCCGGATCTCATGGTTGCTGAACATTAGGGT |
| **For Production of chalcone in *E. coli* cells** | |
| MCS1-CHS-F | ACCATCATCACCACAGCCAGGATCCGATGTCAAAGATTGAGGAGA |
| MCS1-CHS-R | AGGCGCGCCGAGCTCGAATTACCATCAATGGCCACACTCCT |
| MCS2-CHIL-F | ATAAGAAGGAGATATACATAATGGAGTCGAAGATGATCAT |
| MCS2-CHIL-R | TATCCAATTGAGATCTGCCATAGCGTCCGATAACATTAATC |
| pET-4CL-CHS-CHIL1 | CAGGTCGACAAGCTTGCTTAAATAGGCGCCAGCAACCG |
| pET-4CL-CHS-CHIL2 | CTTAAGCATTATGCGGCCGCTCATCGTCATCCTCGG |
| Nru-CHI1 | GGCCATCCAGCCTCGCGTCGGCATGCAAGGAGATGGCGCCC |
| Nru-CHI2 | GGCTACGTCTTGCTGGCGTTCGGGCTACGTCTTGCTGGCGTTCG |
